# Time to lost to follow-up and its predictors among adult patients receiving antiretroviral therapy retrospective follow-up study Amhara Northwest Ethiopia

**DOI:** 10.1038/s41598-022-07049-y

**Published:** 2022-02-21

**Authors:** Animut Takele Telayneh, Mulugeta Tesfa, Wubetu Woyraw, Habtamu Temesgen, Nakachew Mekonnen Alamirew, Dessalegn Haile, Yilkal Tafere, Pammla Petrucka

**Affiliations:** 1 Department of Public Health, Debre Markos University, P.O. Box 269, Debre Markos, Ethiopia; 2 Department of Human Nutrition, Debre Markos University, P.O. Box 269, Debre Markos, Ethiopia; 3 Department of Nursing, Debre Markos University, P.O. Box 269, Debre Markos, Ethiopia; 4 College of Nursing, University of Saskatchewan, Saskatoon, Canada; 5 School of Life Sciences and Bioengineering, Nelson Mandela African Institute of Science and Technology, Arusha, Tanzania

**Keywords:** Infectious diseases, Public health, Therapeutics

## Abstract

Antiretroviral therapy lowers viral load only when people living with HIV maintain their treatment retention. Lost to follow-up is the persistent major challenge to the success of ART program in low-resource settings including Ethiopia. The purpose of this study is to estimate time to lost to follow-up and its predictors in antiretroviral therapies amongst adult patients. Among registered HIV patients, 542 samples were included. Data cleaning and analysis were done using Stata/SE version 14 software. In multivariable Cox regression, a p-value < 0.05 at 95% confidence interval with corresponding adjusted hazards ratio (AHR) were statistically significant predictors. In this study, the median time to lost to follow-up is 77 months. The incidence density of lost to follow-up was 13.45 (95% CI: 11.78, 15.34) per 100 person-years. Antiretroviral therapy drug adherence [AHR: 3.04 (95% CI: 2.18, 4.24)], last functional status [AHR: 2.74 (95% CI: 2.04, 3.67)], and INH prophylaxis [AHR: 1.65 (95% CI: 1.07, 2.56) were significant predictors for time to lost to follow-up. The median time to lost was 77 months and incidence of lost to follow-up was high. Health care providers should be focused on HIV counseling and proper case management focused on identified risks.

## Introduction

Human Immunodeficiency Virus (HIV) which potentially leads to Acquired Immune Deficiency Syndrome (AIDS) is a global health problem^1–4^. Globally, an estimated 37.7 million (30.2 million–45.1 million) people were living with HIV and around 4,000 new infections every day, 2020^5,6^. Africa, Asia, and Latin America were the major affected continents by HIV infection^5,7,8^. In 2020, more than 680, 000 deaths and destroyed 21.5 US dollars for ADIS response in low and middle-income countries^6^. Anti-Retroviral Therapy (ART) lowers viral load only when people living with HIV (PLHIV) fully adhere to the treatment regimen continuously for life^3,9–11^. However, the viral suppression rate was 66% globally, 59% in western and central Africa, and 72% in Ethiopia in 2020^5,6^. Optimal HIV care retention is very crucial to address HIV global 2030 goals^5,12^.

Lost to follow-up is defined as a patient who has not received ART medication for more than 30 days of their last missed drug collection appointment^8,13–15^. Lost to follow-up is the persistent major challenge to the success of the ART programs in low-resource settings including Ethiopia^2,8,10,16,17^. This may increase treatment failure (i.e. clinical, immunological, and Virological), morbidity, mortality, and drug resistance^7,8,10^. The patient retention status is an important measurement of ART program effectiveness^7,10^. In the previous finding in Asia, the trend of lost to follow-up among HIV- positive patients’ received ART was 9%^18^. In sub-Saharan Africa, public sector HIV treatment clinics five years retrospective follow-up revealed that 24.6% were lost to follow-up^10^. The proportion of lost to follow-up was found to range in different settings from 16.4%^19^ in South Africa, 28% in Nigeria, 12.4%^20^ in Malawi, and 3% in seven teaching hospitals, 11% in Wukro Hospital in Tigray Region, Ethiopia, and 21.3% in Oromia Region, Ethiopia^2,9,15,21–23^. In resource-limited settings of West Africa, males had a 14% higher rate of lost than females. The incidence rate of lost to follow-up was 9.2/100 person-years (PYs)^23^ in a large-scale ecological study in Sub-Saharan Africa. Other studies found rates of 12.8%/100 PYs^15^ in South Africa, 11.6/100PYs^24^ in Pawi General Hospital in Ethiopia, 8.2/100 PYs in Aksum Hospital^13^, and 12.26/100 PYs^25^ in Gondar Specialized Comprehensive Hospital in Ethiopia. The median time to lost to follow-up was three months, and 14% of them had lost in the first day of antiretroviral therapy^26^.

The findings in Nigeria showed the incidence rate of treatment interruption was highest in the first six months of ART at 18.2/100 PYs but decreased after two years to 8.8/100 PYs. Lost to follow-up from treatment increased by 1.30 times for each calendar year in a study in South Africa^15^. Previous existing evidence revealed, patients who had hemoglobin (Hgb) markers, nutritional deficiencies, opportunistic infections, cancers, illiterate, unmarried, rural dweller, alcohol drank, tobacco smoking, CD4 level, WHO clinical stage, short HIV infection history, age, drug side effect, viral load, employment status, regimen change, TB infection, male gender, weight, mental illness, receiving INH therapy, functional status, and advanced HIV disease affecting the hematopoietic system were predictors of lost to follow-up^7,8,11,13,24,27–34^.

Previously shared evidence, in Ethiopia, addresses adherence to ART and only a few studies were conducted to assess predictors of lost to follow-up. However, the data regarding time to lost to follow-up after ART initiation, incidence lost follow-up, and predictors were unsaturated. Therefore, this study aims to estimate the time to lost to follow-up and its predictors among adult patients receiving antiretroviral therapy in Amhara, Northwest Ethiopia.

## Results

### Socio-demographic characteristics of the participants

A total of 542 participants were included in the study. More than half (60.5%) of participants were females. The mean age of participants was 34.34 ± 10.49 years standard deviation. Two hundred sixty-six (49%) patients were married and 66 (12.2%) of the partners were known to be HIV-positive. In this study, two-third (66.8%) of study participants had disclosed their HIV-positive status. Almost one-fourth (27.5%) of study subjects had used family planning during the study period, with 37 (6.8%) of them using natural family planning methods and only 29 (8.8%) study participants had become pregnant in this follow-up period (Table 1).

**Table 1:** Socio-demographic characteristic of adult HIV patients in Bichena health center, North West, Ethiopia, January 1, 2008–December 30, 2017.

Variables	Outcome		Total N (%)
	Event N (%)	Censored N (%)	
**Sex**			
Male	84 (38)	130 (40.49)	214 (39.50)
Female	137 (62)	191 (59.51)	328 (60.50)
**Age**			
15–24 years	31 (14.03)	43 (13.40)	74 (13.60)
25–34 years	85 (38.46)	138 (43)	223 (41)
35–44 years	66 (29.86)	88 (27.41)	154 (28.40)
45–54 years	28 (12.67)	36 (11.21)	64 (12)
≥ 55 years	11 (4.98)	16 (4.98)	27 (5)
**Marital status**			
Single	27 (12.22)	32 (9.96)	59 (10.90)
Married	194 (87.78)	289 (90.04)	483 (89.10)
**Educational status**			
No formal education	142 (64.25)	207 (64.49)	349 (64.50)
Primary education (1–8 grades)	37 (16.75)	70 (21.80)	107 (19.70)
Secondary education (9–10 grades)	27 (12.21)	33 (10.28)	60 (11)
High school and above education	15 (6.79)	11 (3.43)	26 (4.80)
**Region**			
Amhara	216 (97.74)	316 (98.44)	532 (98.20)
Others (Oromo and Tigray)	5 (2.26)	5 (1.56)	10 (1.80)
**Occupation**			
Unemployment	37 (16.74)	57 (17.75)	94 (17.30)
Employed	184 (83.26)	264 (82.25)	448 (82.70)
**Residence**			
Within the catchment	147 (66.51)	183 (57)	330 (60.90)
Out of the catchment	74 (33.49)	138 (43)	212 (39.10)
**Number of children (322)**			
≤ 4 children	125 (93.98)	174 (92.06)	299 (92.90)
> 4 children	8 (6.02)	15 (7.94)	23 (7.10)
**Disclosure status of HIV**			
No	72 (32.58)	108 (33.64)	180 (33.20)
Yes	149 (67.42)	213 (66.36)	362 (66.80)

### Medical history, clinical, and drug adherence characteristics

In this study, 66 (12%) and 105 (19.4%) of participants had a history of diarrhea and fever respectively. Two hundred (36.9%) study participants had a history of opportunistic infection and 83 (15.3%) of participants had taken INH preventive therapy. Only (53%) of study participants had Hgb tested during their enrolment. The median CD4 level of study participants was 208cells/mm^3^ with IQR (106–328.25) (Tables 2 and 3).

**Table 2:** Past medical history and drug adherence characteristic of adult HIV patients in Bichena health center, Amhara, North West, Ethiopia, January 1, 2008–December 30, 2017.

Variables	Outcome		Total N (%)
	Event N (%)	Censored N (%)	
**Opportunistic infection**			
No	142 (64.25)	200 (62.30)	342 (63.10)
Yes	79 (35.75)	121 (37.70)	200 (36.90)
**TB treatment**			
No	191 (89.60)	295 (91.90)	486 (89.70)
Yes	30 (10.40)	26 (8.10)	56 (10.30)
**TB treatment regimen (56)**			
2RHZE/6EH	6 (20)	16 (61.54)	22 (39.30)
2RHZE/4RH	24 (80)	10 (38.46)	34 (59.70)
**Cotrimoxazole therapy**			
No	37 (16.74)	58 (18.07)	95 (17.50)
Yes	184 (83.26)	263 (81.93)	447 (82.50)
**INH prophylaxis**			
No	198 (89.59)	261 (81.31)	459 (84.70)
Yes	23 (10.41)	60 (18.69)	83 (15.30)
**ART drug side effect**			
No	202 (91.40)	299 (93.15)	501 (92.40)
Yes	19 (8.60)	22 (6.85)	41 (7.60)
**TB screening done**			
No	185 (83.71)	268 (83.49)	453 (83.60)
Yes	36 (16.29)	53 (16.51)	89 (16.40)
**ART drug adherence**			
Good	148 (66.97)	276 (85.98)	424 (78.20)
Fair	15 (6.79)	16 (4.98)	31 (5.70)
Poor	58 (26.24)	29 (9.03)	87 (16.10)
**Risk behavior**			
No	119 (53.85)	206 (64.17)	325 (60)
Yes	102 (46.15)	115 (35.82)	217 (40)

**Table 3:** Clinical and laboratory characteristic of adult HIV patients in Bichena health center, Amhara, North West, Ethiopia, January 1, 2008–December 30, 2017.

Variables	Outcome		Total N (%)
	Event N (%)	Censored N (%)	
**Last functional status**			
Working	104 (47.06)	265 (82.56)	369 (68.10)
Bedridden	50 (22.62)	14 (4.36)	64 (11.80)
Ambulatory	67 (30.32)	42 (13.08)	109 (20.10)
**Baseline WHO stage**			
I & II	98 (44.34)	136 (42.37)	234 (43.20)
III & IV	123 (55.66)	185 (57.63)	308 (56.80)
**Baseline nutrition screened done**			
No	5 (2.23)	6 (1.87)	11 (2)
Yes	216 (97.77)	315 (98.13)	531 (98)
**Baseline BMI**			
Underweight	84 (38)	126 (39.25)	210 (38.70)
Normal	130 (58.83)	184 (57.32)	314 (58)
Overweight	7 (3.17)	11 (3.43)	18 (3.30)
**Baseline CD4**			
≤ 100cells/mm^3^	63 (28.51)	57 (17.76)	120 (22.20)
101–200cells/mm^3^	68 (30.77)	66 (20.56)	134 (24.70)
≥ 201cells/mm^3^	90 (40.72)	198 (61.68)	288 (53.10)
**Baseline regimen**			
D4T base	29 (13.12)	49 (15.26)	78 (14.50)
AZT base	59 (26.70)	104 (32.40)	163 (29.50)
TDF base	133 (60.18)	168 (52.34)	301 (56)
**Regimen change**			
No	202 (91.40)	280 (87.23)	482 (89)
Yes	19 (8.60)	41 (12.77)	60 (11)

### Lost follow-up status and treatment outcome

Of the 542 study participants, 221 (40.8%) (95% CI: 36.69, 44.98) were lost to follow-up, nearly one-third (32.5%) were on active treatment, 20.1% were transferred out, and 6.6% died in this retrospective follow-up cohort.

**Figure 1:**
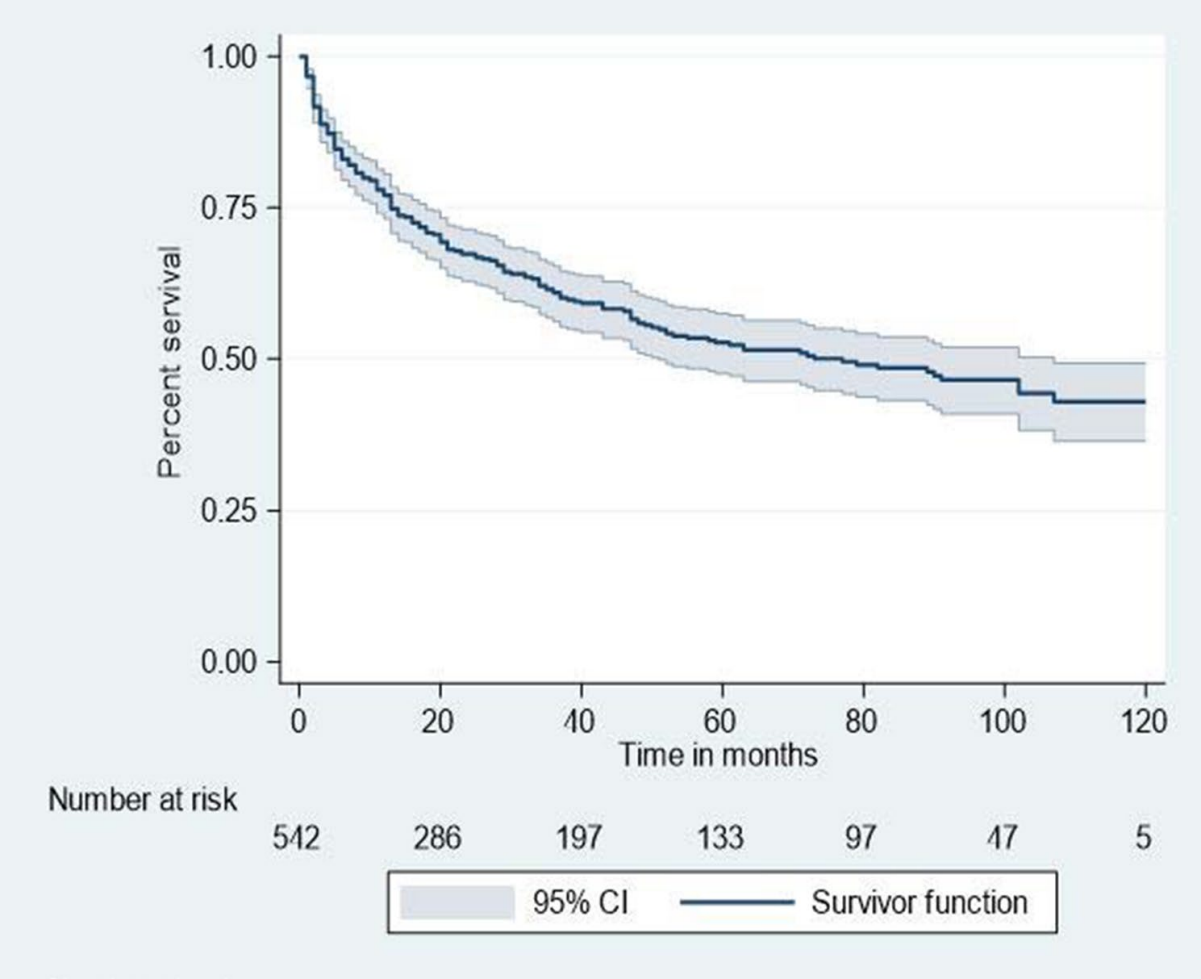
Overall survival estimates among HIV patients in Bichena health center, Amhara, North West Ethiopia, January 1, 2008–December 30, 2017.

The incidence density of lost to follow-up was 13.45 (95% CI: 11.78, 15.34) per 100 PYs. In this finding, half of the patients had lost to follow-up at 77 months follow-up (95% CI: 51, 107) period (Fig. 1).

The incidence rate of lost to follow-up is quite different based on duration point in treatment as 3.32/100 person-months at first month, 5.20/100 person-months at second month, 3.11/100 person-months at 3 months, and 2.20/100 person-months at 6 months after ART treatment initiation. Even though the incidence density in the first-year follow-up is high, it shows a general pattern of declines over the first-six years follow-up as 27.41/100PYs at 1st year, 13.87/100PYs at 2nd year, 9.82/100PYs at 3rd year, 8.45PYs at 4th year, 6.04PYs at 5th year, and 4.18PYs at 6th year. Controversially, the incidence of lost to follow-up was high, 7.3/100PYs at the end of the 9th year follow-up.

### Predictors of time to lost to follow-up among adult HIV patients

**Figure 2:**
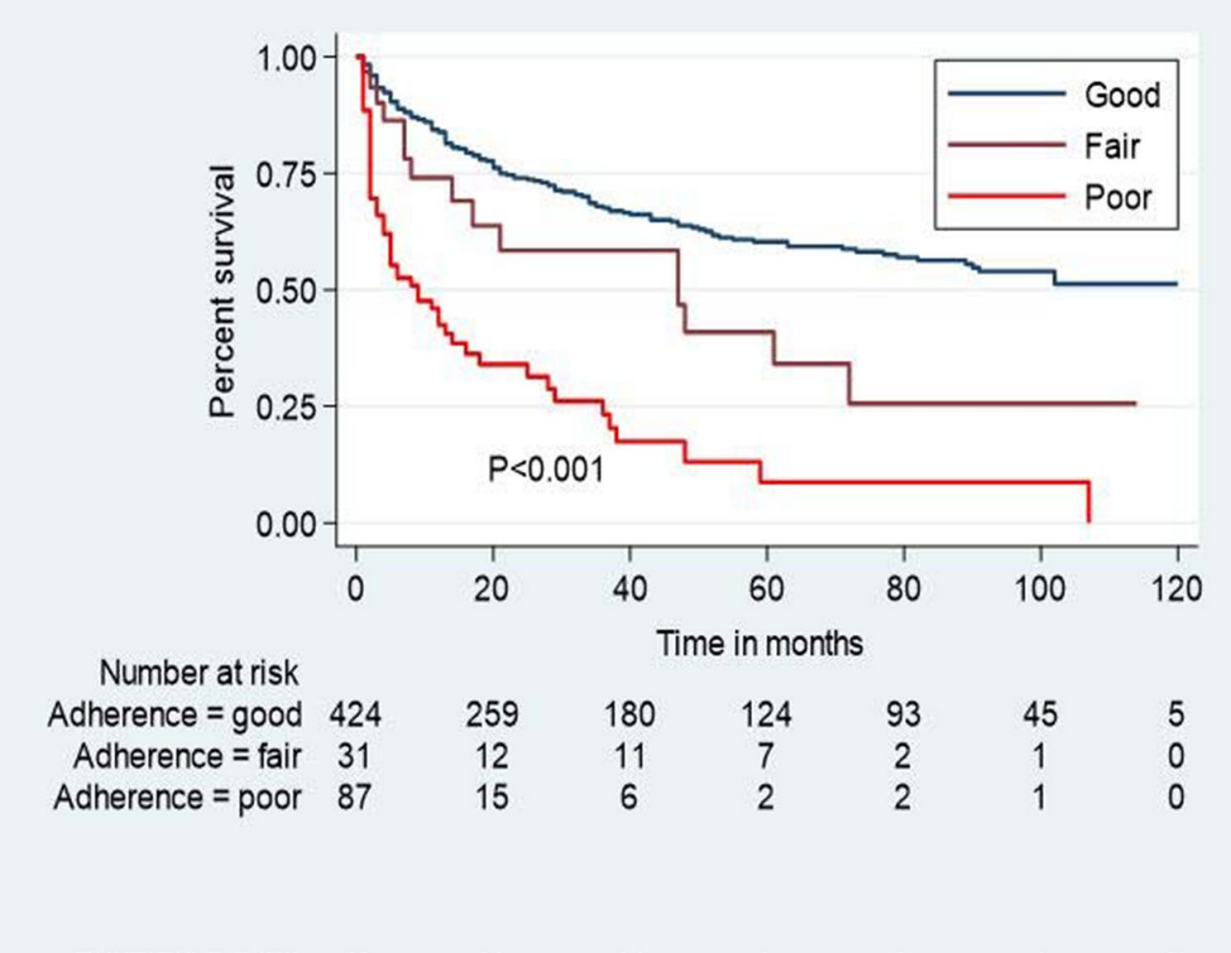
Kaplan–Meier survival estimates among HIV patients in Bichena health center, Amhara, North West, Ethiopia, January 1, 2008–December 30, 2017 by ART drug adherence level.

**Figure 3:**
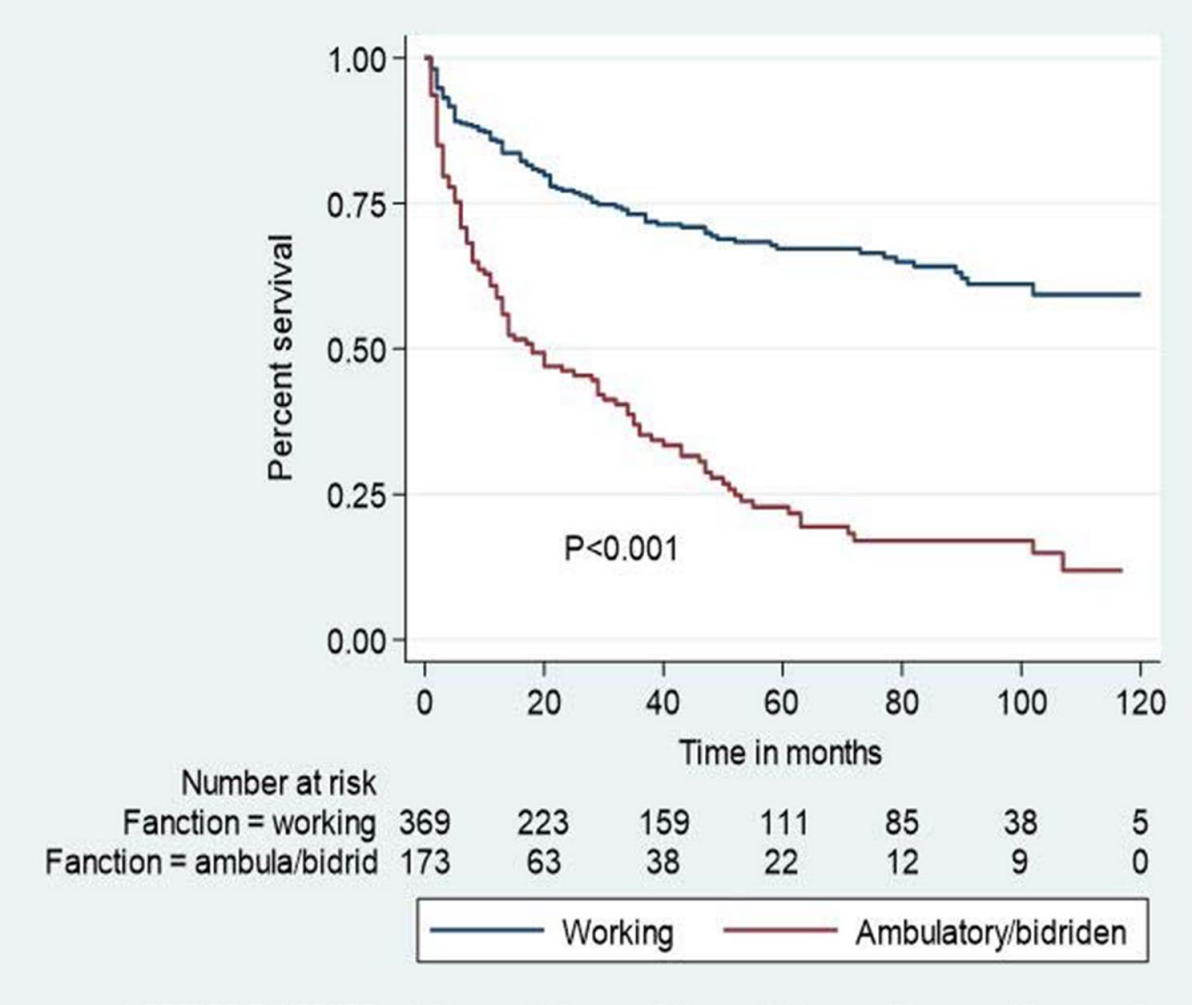
Kaplan–Meier survival estimates among HIV patients in Bichena health center, Amhara, North West, Ethiopia, January 1, 2008—December 30, 2017, by last functional status.

In multivariable Cox regression analysis, statistically significant variables were selected. Patients who had poor ART drug adherence were three times more at risk of lost to follow-up compared to the cohort with good ART drug adherence [AHR: 3.04 (95% CI: 2.18, 4.24)]. Similarly, those who had ambulatory or less last functional status were 2.74 times more at risk to lost to follow-up compared to working last functional status [AHR: 2.74 (95% CI: 2.04, 3.67)]. Moreover, patients not receiving INH prophylaxis therapy were 1.65 times more at risk to lost to follow-up compared to counterparts taking INH therapy [AHR: 1.65 (95% CI: 1.07, 2.56)] (Table 4 and Figs. 2, 3 and S2).

**Table 4:** Bi-variable and multi-variable analysis of time to lost to follow up and its predators among adult HIV patients in Bichena health center, Amhara, North West, Ethiopia, January 1, 2008–December 30, 2017.

Variable	Outcome		CHR with 95%CI	AHR with 95%CI
	Event N (%)	Censor N (%)		
**Baseline CD4**				
< 200cells/mm^3^	131 (59.28)	123 (38.32)	1.66 (1.27, 2.17)	1.10 (0.82, 1.48)
≥ 200cells/mm^3^	90 (40.72)	198 (61.68)	1	1
**ART drug adherence**				
Good	148 (66.97)	276 (85.98)	1	1
Fair	15 (6.79)	16 (4.99)	1.85 (1.09, 3.15)	1.42 (0.82, 2.44)
Poor	58 (26.24)	29 (9.03)	4.48 (3.27, 6.15)	3.04 (2.18, 4.24)*
**Last functional status**				
Working	104 (47.05)	265 (82.55)	1	1
Ambulatory and less	117 (52.95)	56 (17.45)	3.46 (2.65, 4.52)	2.74 (2.04, 3.67)*
**TB treatment**				
No	191 (86.43)	295 (91.90)	1	1
Yes	30 (13.57)	26 (8.10)	1.38 (0.94, 2.02)	1.09 (0.73, 1.62)
**INH prophylaxis**				
No	198 (89.59)	261 (81.31)	1.95 (1.27, 3.01)	**1.65 (1.07, 2.56)**
Yes	23 (10.41)	60 (18.69)	1	1
**Risk behavior**				
No risk	119 (53.85)	206 (64.17)	1	1
Risk behavior	102 (46.15)	115 (35.83)	1.26 (0.97, 1.65)	1.20 (0.92–1.57)
**Cotrimoxazole therapy**				
No	37 (16.74)	58 (18.07)	1.37 (0.96, 1.95)	1.34 (0.93–1.92)
Yes	184 (83.25)	263 (81.93)	1	1
**Disclosure status**				
No	72 (32.58)	108 (33.64)	1.30 (0.98, 1.74)	1.17 (0.87–1.56)
Yes	149 (67.42)	213 (66.36)	1	1

*p < 0.001.

## Discussion

This study was designed to estimate time to lost to follow-up among adult HIV/AIDS patients. ART declines HIV/AIDS-related death, hence high retention in ART care is required to optimize treatment outcomes^2,23,34,35^. In this study, the overall incidence density of lost to follow-up was 13.45 (95% CI: 11.78, 15.34) per 100 PYs. The study finding is similar to previous evidence with 12.26/100PYs^25^ in Gondar Specialized Hospital in Ethiopia and 12.8/100PYs^15^ in South Africa. The current finding is greater than the 10.9/100 PYs in Gondar Specialized Hospital Ethiopia^34^, 11.6/100 PYs in Pawi Hospital (Northwest Ethiopia)^24^, and 9.2/100 PYs in a large scale ecological study in sub-Saharan Africa^23^. The possible reason for this variation might reflect differences in the study period, settings, sample size, adherence problem, drug-related side effects, and counseling barriers. As evidence revealed, the quality of clinical service care was different between different levels of health facilities this may have a role on lost to follow-up^36^. This study was conducted in a single health center that has poor health care service (such as lack of medical equipment, inadequate laboratory investigations, poor case management, lack of senior clinicians, and high turnover rate of health professionals), poor health-seeking behaviors, lengthy travel distances to get health service, and low literacy levels this might be increase likely hood of lost to follow-up. Even though limited evidence existed in Ethiopia that was conducted at the hospital level, this study setting difference between hospital and health center might introduce variation service quality and level of care. There is also outcome measurement variation, one month and more missed in this study may increase the incidence. Unlikely, this evidence was lower than findings 21.4/100PYs in Asia–Pacific regions^28^. This difference may be due to variation of outcome measurement lost to follow-up as used patient not seen in the clinic more than 12 months.

The incidence rate of lost to follow-up is different across time with 3.32/100 person-months at the first month, 5.20/100 person-months at the second month, 3.11/100 person-months at 3 months, and 2.20/100 person-months at 6 months follow-up after ART treatment initiation. Although the incidence rate in the first-year follow-up (27.41/100 PYs) is high, it shows a slight decline over the first-six years follow-up with 13.87/100 PYs at 2nd year, 9.82/100 PYs at 3rd year, 8.45 PYs at 4th year, 6.04 PYs at 5th year, and 4.18 PYs 6th year. Despite this, the incidence was increased to 7.3/100 PY at the end of 9th-year follow-up. This finding is consistent with previous findings from Tigray, Southern, and Oromia regions in Ethiopia, and Nigeria^21,36–38^. However, this finding lies in disagreement with a South African study^15^. The patients who stayed long periods on ART have had improved adherence, relief from drug-related side effects, less likelihood of opportunistic infections, progressively improved ART service quality in the facility, and establishing lost to follow-up tracing modalities by adherence supporters and volunteer networks in the community. This might have a direct effect on patient retention on antiretroviral therapy.

The proportion of lost to follow-up is 40.8% in this study which is higher than previous reports which ranged from 2.87 to 25.3% in various regions of Ethiopia^2,22,24,27,34,39–42^, 12.4% in Malawi^20^, 16.4% in South Africa^19^, 28% in Nigeria^21^, 24.6% in Sub-Saharan Africa^10^, and 243.9% in South-Eastern Nigeria Hospitals^30^. The possible explanation for this disparity may be settings (in this study, i.e. laboratory service, counseling, and clinical case management), the difference in health center compared to most of the previous findings in hospitals, and poor health-seeking behavior, used measurement difference lost to follow-up, socioeconomic, and socio-demographic differences across facilities and countries impacting on lost to follow-up.

ART drug adherence was a statistically significant predictor of lost to follow-up; patients who had poor ART drug adherence were 3 times more at risk of lost to follow-up compared to good ART drug adherence. This is consistent with the study conducted in Gondar Comprehensive Specialized Hospital, Oromia region, Jima Specialized Hospital Ethiopia, sub-Saharan African, low and middle-income countries meta and systematic analysis, and Nigeria^2,4,21,23,25,32^. The possible reasons may be due to HIV patients’ hopelessness on treatment, conflicts with religious concerns, demanding traditional healers, lack of social support, fear of stigma and discrimination, drug side effects, and other socio-economic reasons that cause them to miss their medication.

Similarly, last functional status is statistically significant to lost to follow-up: Patients who had ambulatory and lower last functional status were 2.74 times more at risk to lost to follow-up compared to those with working as last functional status. This study finding is consistent with the studies conducted in Kimbata and Hadiya zone, Oromia region, Jinka hospital Ethiopia, and (sub-Saharan Africa, low and middle-income countries) meta and systematic analysis,^2,8,10,22,25,32,43^. The possible reason for this finding might be ambulatory and less functional status patients were more likely to experience lost to follow-up due to inability to work or decrease productivity in all aspects of social, economic, and financial influences. They also need help and close supervision to ensure adherence to ART, which might have been a high probability to the drug side effects; hence, collectively these experiences may affect retention on ART care.

Moreover, those who did not take INH prophylaxis therapy were found to be 1.65 times more at risk to lost to follow-up compared to counterparts who did take INH therapy. This finding supported previous findings in Gondar Comprehensive Specialized Hospital, Pawi General Hospital, Wukro Hospital, public hospitals Binishangul Gumuz, and in southern Ethiopia^24,25,34,37,40,41^. INH prophylaxis therapy recommended by the national treatment guideline may direct effect on decrease lost to follow-up by strengthening retention of HIV patients on ART service since INH prophylaxis therapy controls one of the most common life-threatening co-morbidity and mortality threats (i.e., tuberculosis co-infection) among HIV infected people^3^. This existing intervention to improve the health of the patient may encourage patients’ retention on ART care.

In conclusion, the time to lost half of the patients was 77 months, and the incidence of lost to follow-up was high. Poor drug adherence, ambulatory & less last functional status, and not taken INH prophylaxis were predictors for lost to follow-up among adult HIV/AIDS patients. The government gives special attention to the quality of HIV care at the health center level. There is an imperative to design new initiatives to reduce lost follow-up at the national level including improving ART accessibility modalities, early reminder strategies through tell-medicine, and expansion of laboratory services at preferred sites.

## Methods

### Study design and setting

An institution-based retrospective follow-up study was conducted from January 1, 2008 to December 30, 2017. Data were collected from February 28–March 16, 2018. The study was conducted at the Bichena Health center. It is found 355 km (km) from Bahir Dar city of Amhara National Regional State and 265 km from Addis Ababa capital of Ethiopia. The health center started ART services in 2008, serving a 51,653 catchment population. It was the first high load ART site health center in the East Gojjam Zone, having 2,770 patients registered for ART service, among those 2,655 (95.8%) were adult ART users, which constituted the source population in this study.

### Participants

**Figure 4:**
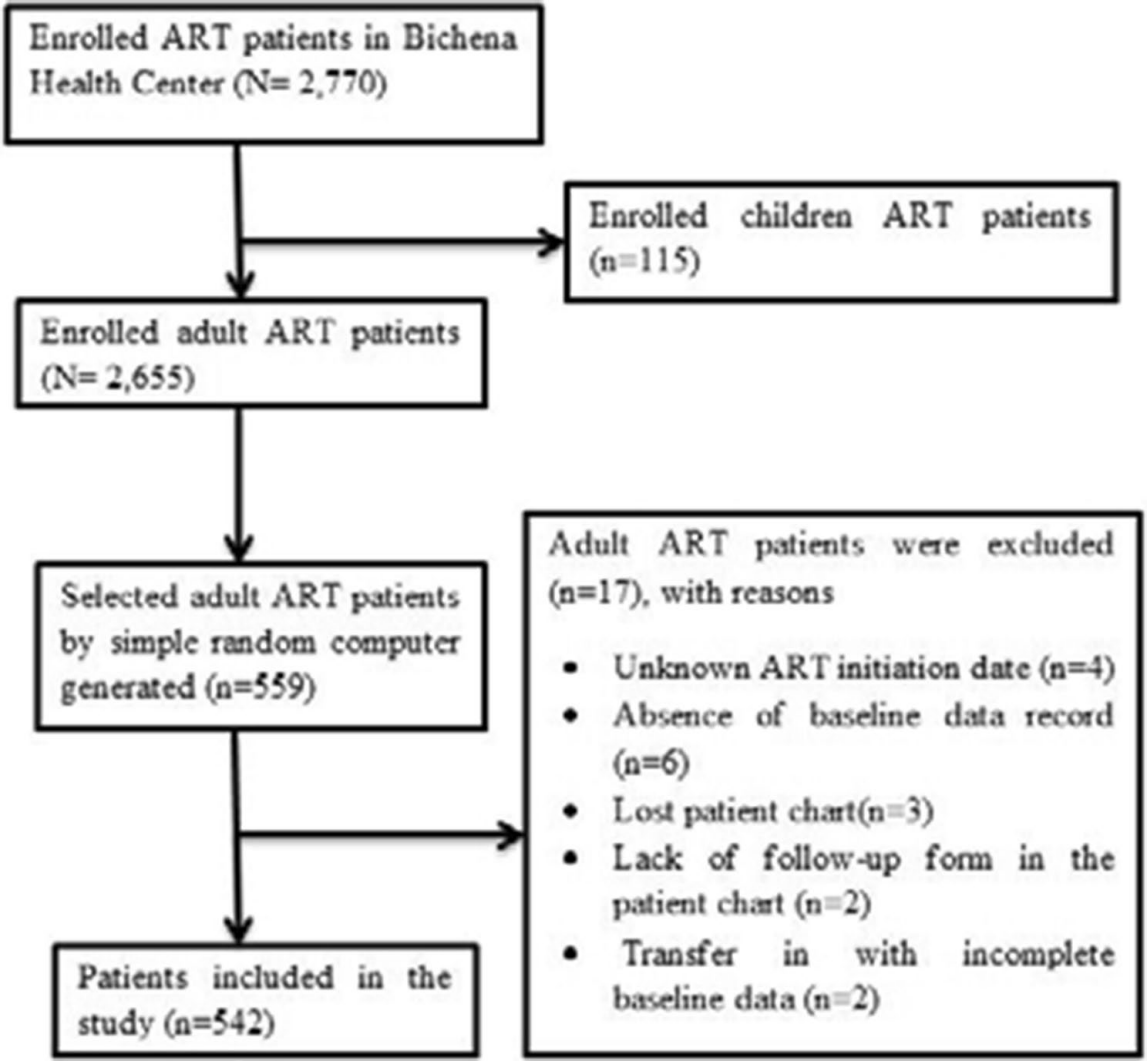
A flow chart to show the selection of study participants among HIV patients in Bichena health center, Amhara, January 1, 2008–December 30, 2017.

The study population was all HIV-infected adults (> 15 years) who enrolled at Bichena Health Center ART clinic from January 1, 2008–December 30, 2017. Adult HIV-infected patients who had at least-one ART follow-up visit were eligible to include. Those patients who had unknown ART initiation date, absence of baseline data record or follow-up form in the patient chart and transfer in with incomplete baseline data were excluded in this study (Fig. 4).

### Definition of variables

- Lost to follow-up: A patient who has not received ART medication for more than 1 month of their last missed drug collection appointment^8,13–15^.
- Disclosure: If anyone knows the status of the patient/child at the workplace, school, family, and other community members^44^.
- Adherence: The level of adherence was assessed at every time the patient comes to clinical visit as good (> 95%) missed ≤ 2 doses in 30 doses or ≤ 3 doses of 60 doses, fair (85–94%) missed 3–5 doses in 30 doses or 4–8 doses of 60 doses, and poor (< 85%) missed ≥ 6 doses in 30 doses or ≥ 9 doses of 60 doses^44^.
- Risk behavior: If the patient has one or more of the following risks (i.e. having multiple sexual partners, practicing unsafe sex, use of tobacco, and alcohol drink) were considered as a risk behavior^44^.
- Functional status: The functional status was assessed continuously to every clinical visit as (working = able to perform usual work in or out of the house, harvest, go to school, ambulatory = ambulatory but not able to work or able to perform activities of daily living, and bedridden = not able to perform activities of daily living)^44^.
- Regimen change: As an event, through the follow-up period was ascertained retrospectively when the patients are recorded as changed their regimen and started other ART drugs^45^.
- TB screening: The screening is done at the time of ART initiation and every clinical visit what every the screening is through using x-ray or clinically but not on any anti-TB medication^44^.

### Sample size determination and sampling procedure

The sample size was calculated by a double population formula using Epi Info™ version 7 software considering the following assumptions: level of precision 5%, confidence interval 95%, power 80%, the proportion of unexposed (female gender) 22.87%^15^, the proportion of exposed (male gender) 28.60%^15^, hazard ratio 1.51, and nonresponse rate 10%. Hence, a sample size of 559 was computed with the inclusion of a 10% non-response rate. The study participants were selected by computer-generated simple random sampling technique using their medical record numbers.

### Data collection procedure and quality assurance

A retrospective record review technique was used to collect the required data. All eligible medical recorded data were taken from the ART intake forms and follow-up charts. The data was collected by two diploma nurses and one public health officer as supervisor. The checklist was adapted from the standard Ministry of Health ART intake forms and follow-up charts. Socio-demographic data, past medical history, drug adherence, and clinical & laboratory results were the components of the checklist. Data quality was maintained starting during designing the checklist, training for data collectors’ ad supervisor, and close supervision held during the entire data collection period.

### Data processing and statistical analysis

EpiData version 4.2 Entry client was used for data entry and Stata/SE version 14.0 was used for data cleaning and analysis. Data were checked for completeness and inconsistencies before the analysis. Survival Kaplan Meier estimator was used to show the survival and failure estimate curve. Cox regression analysis was fitted to identify the association between dependent and independent variables. Model fitness was checked by Cox proportion hazard model, log–log plot graphically, and test proportional hazard assumptions by Scheonfeld residual. The goodness of fit was checked by running Cox-Snell residual analysis (Fig. S1). Descriptive analysis; frequency tables, mean, median, range, and graphs were done to describe important variables. All predictors that had a significant association with lost to follow-up in the bivariable Cox regression model with p-value < 0.25 were included in multivariable analysis. Finally, significant predictors at p-value < 0.05 in multivariable Cox regression at 95% CI were declared statistically significant for lost follow-up.

### Ethics statement and consent to participate

This study was approved by the Debre Markos University, College of Health Science ethical review committee (#HSC/R/C/S/P/Co/696//11/10). The need of informed consent was waived by the Ethics committee of Debre Markos University. The information given was maintained strictly confidential and used for this study purpose only. All procedures were carried out in accordance with relevant guidelines and regulations.

### Limitations

In this study, about 17 patient charts for those who had not or incomplete baseline data were not included. Since we are used secondary data important predictors like viral load, hemoglobin test, and other clinical factors were not included as they were not properly recorded. Also, the lost follow-up might be affected by length–time bias. Thus, this finding should be considered with these limitations.

## Supplementary Information

Supplementary Figures.

## Data Availability

The datasets used and/or analyzed during the current study are available from the corresponding author on reasonable request.
