# Supplementary material for: Time to lost to follow-up and its predictors among adult patients receiving antiretroviral therapy retrospective follow-up study Amhara Northwest Ethiopia
Source: Sci Rep. 2022 Feb 21;12:2916. doi: 10.1038/s41598-022-07049-y (PMC8861049; doi:10.1038/s41598-022-07049-y)
Supplement: Supplementary file 1 — Supplementary Figures. [file 41598_2022_7049_MOESM1_ESM.pdf]

Time to lost to follow-up and its predictors among adult patients receiving antiretroviral therapy retrospective follow-up study Amhara Northwest Ethiopia

Animut Takele Telayneh[1*], Mulugeta Tesfa[1], Wubetu Woyraw[2], Habtamu Temesgen[2], Nakachew Mekonnen Alamirew[1], Dessalegn Haile[3], Yilkal Tafere[1], and Pammla Petrucka[4, 5]

[1]Department of Public Health, Debre Markos University, Debre Markos, Ethiopia, P.O. Box 269

[2]Department of Human Nutrition, Debre Markos University, Debre Markos Ethiopia, P.O. Box 269

[3]Department of Nursing, Debre Markos University, Debre Markos, Ethiopia, P.O. Box 269

[4]College of Nursing, University of Saskatchewan, Saskatoon, Canada

[5]School of Life Sciences and Bioengineering, Nelson Mandela African Institute of Science and Technology, Arusha, Tanzania

ATT: animuttakele@gmail.com MT: mulutesfa.g@gmail.com WW: wubetu662@gmail.com HT:habtamutem@gmail.comNMA:nakachewmeku@gmail.com DH: dessalegnhaile@gmail.com YT: yilkal2007@gmail.com PP: pammla.petrucka@usask.ca

* Corresponding author

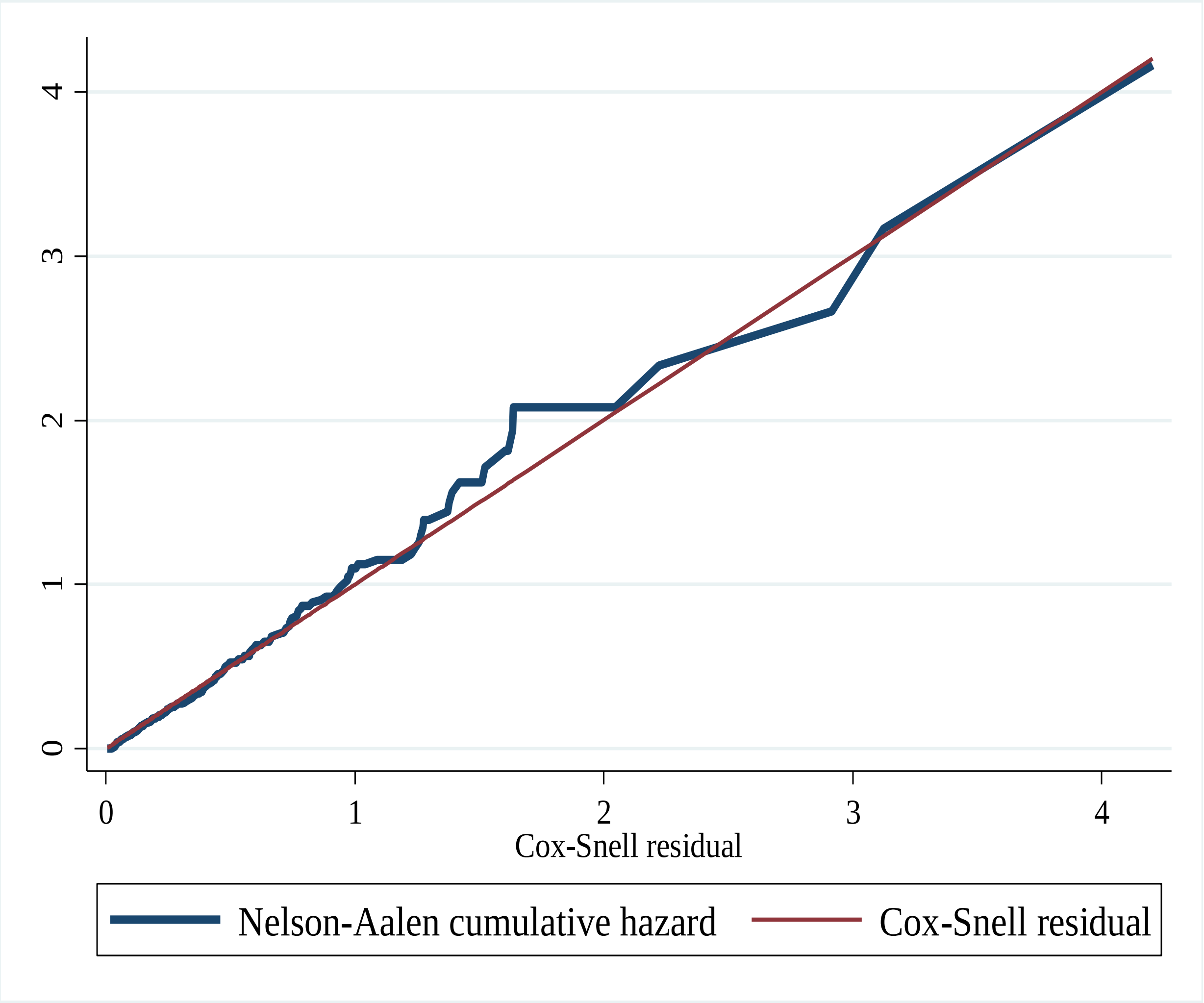

Figure S1: Scheonfeld residual test of proportional hazard assumptions for final model fitness

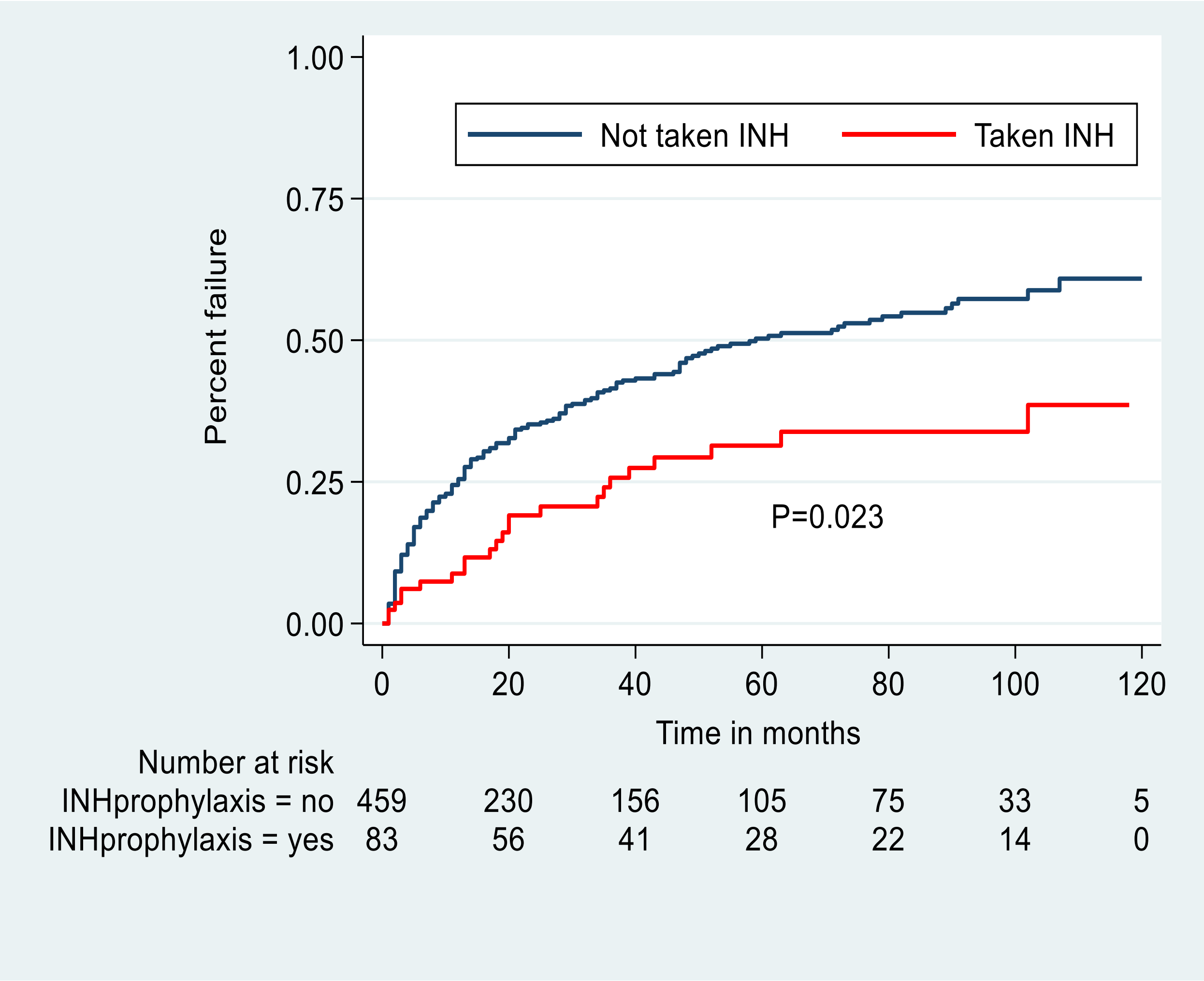

Figure S2: Kaplan-Meier failure estimates among HIV patients in Bichena health center Amhara, North West, Ethiopia, January 1, 2008 - December 30, 2017, by INH prophylaxis intake status
